# Preparation of Hydrophobic Film by Electrospinning for Rapid SERS Detection of Trace Triazophos

**DOI:** 10.3390/s20154120

**Published:** 2020-07-24

**Authors:** Fei Shao, Jiaying Cao, Ye Ying, Ying Liu, Dan Wang, Xiaoyu Guo, Yiping Wu, Ying Wen, Haifeng Yang

**Affiliations:** The Education Ministry Key Lab of Resource Chemistry, Shanghai Key Laboratory of Rare Earth Functional Materials, Shanghai Municipal Education Committee Key Laboratory of Molecular Imaging Probes and Sensors and Department of Chemistry, Shanghai Normal University, Shanghai 200234, China; sf3110@163.com (F.S.); C18360861958@163.com (J.C.); yingye@shnu.edu.cn (Y.Y.); hzlxm2646@163.com (Y.L.); 18817289115@163.com (D.W.); gxy2012@shnu.edu.cn (X.G.); yipingwu@shnu.edu.cn (Y.W.); ying.wen@shnu.edu.cn (Y.W.)

**Keywords:** electrospun, Ag NPs/poly(styrene-*co*-butadiene), SERS, triazophos

## Abstract

For real application, it is an urgent demand to fabricate stable and flexible surface-enhanced Raman scattering (SERS) substrates with high enhancement factors in a large-scale and facile way. Herein, by using the electrospinning technique, a hydrophobic and flexible poly(styrene-*co*-butadiene) (SB) fibrous membrane is obtained, which is beneficial for modification of silver nanoparticles (Ag NPs) colloid in a small region and then formation of more “hot spots” by drying; the final SERS substrate is designated as Ag/SB. Hydrophobic Ag/SB can efficiently capture heterocyclic molecules into the vicinity of hot spots of Ag NPs. Such Ag/SB films are used to quantitatively detect trace triazophos residue on fruit peels or in the juice, and the limit of detection (LOD) of 2.5 × 10^−8^ M is achieved. Ag/SB films possess a capability to resist heat. As a case, 6-mercaptopurine (6MP) that just barely dissolves in 90 °C water is picked for conducting Ag/SB-film-based experiments.

## 1. Introduction

Surface-enhanced Raman scattering (SERS) spectroscopy, owing to its uncountable merits that provide specific vibrational fingerprint information, is ultrasensitive and noninvasive, and requires less sample preparation, has received broad attention. So far, SERS has been used in the fields of food safety, biological medicine, and environmental monitoring [1,2,3,4,5,6]. For real application purposes, in the literature, various preparation methods for SERS substrates have been extensively explored to optimize the morphologies, compositions, and inter-particle spacing between the neighboring noble metallic nanoparticles [7,8,9]. Additionally, for target molecules to directly or proximately contact the surface or hot spots regions with accurate plasmon resonance fields, it is also a key issue to improve the sensitivity of SERS detection. As a solution, construction of composite SERS substrates by modification of gold or silver NPs in flexible support materials [10,11,12,13,14,15] is regarded.

As is well known, a flexible SERS substrate could capture the analyte by contact [16,17,18,19]. In the literature, SERS films are prepared with printing [20,21,22], dyeing [23], and electrospinning methods [24,25,26]. Of these, electrospinning has attracted favorable concern in virtue of its straightforward procedure. The resultant fibers possess large porosity and high surface-area-to-volume ratios [27,28,29]. Electrospun fiber films are easily modified with SERS-active noble metal nanoparticles, which are expected to generate high SERS signals in a homogeneous way [30]. For instance, Xu et al. constructed a 3D substrate (Ag/Au NR-PCL fibers) for separate SERS testing of three kinds of arsenic species [31]. Zhang et al. reported flexible Ag NPs on the nanofibers by means of seed-mediated growth [32]. In addition, by concentrating NPs to a limited region of a hydrophobic surface, high SERS signals of the target sample were achieved [33]. The electrospun nanofiber membrane-based SERS substrate is hydrophobic, providing a good platform to adsorb organic samples for producing accurate Raman signals [34].

In this work, we employed electrospinning technology to obtain a flexible poly(styrene-*co*-butadiene) (SB) nanofiber film, and then we modified Ag NPs onto the SB film, designated as Ag/SB. SB films with a hydrophobic surface are beneficial to the certain aggregation of Ag NPs without the “coffer ring effect” via a small touching area [33]. As an application case, target organophosphate pesticide residues on the epidermis of fruits and also in juice could be captured by the hydrophobic surface of Ag/SB and come into contact completely with the SERS substrate, after being dried. The Ag/SB SERS-based protocol is propitious for food safety monitoring. Furthermore, Ag/SB films are both rigid like plastic and elastic like rubber, have the ability to resist heat, and could directly detect compounds that are poorly water-soluble in hot water, such as 6-mercaptopuriue (6-MP).

## 2. Experimental Section

### 2.1. Materials

Poly(styrene-*co*-butadiene) and silver nitrate (AgNO_3_, 99.9%) were purchased from Sigma Aldrich, St. Louis, MO, USA. R6G (C_28_H_31_N_2_O_3_C, 95%), triazophos (C_12_H_16_N_3_O_3_PS, 97%), and sodium citrate (Na_3_C_6_H_5_O_7_·2H_2_O, 99.8%) were obtained from Aladdin, Shanghai, China. *N*,*N*-Dimethyl formamide (C_3_H_7_NO, 99.5%) and tetrahydrofuran (C_4_H_8_O, 99.0%) were bought from Merck Chemicals, Mumbai, India. All reagents were of analytical reagent grade and used without further purification. Apples and apple juice were purchased from the local supermarket. All aqueous solutions were prepared by using ultrapure water (18 MΩ·cm).

### 2.2. Characterizations

The surface morphology of the electrospun nanofibers and the final Ag/SB SERS substrate were measured with scanning electron microscopy (SEM, JEOL6380LV). First, SEM samples were sputter-coated with a thin Au layer. For water contact angles (WCAs), measurement of the samples was performed by a contact angle measuring instrument (KRUSS, Hamburg, Germany, DSA30) with the surface tension measurement from 1 × 10^−2^ to 2000 mN·m^−1^. Ag NPs were characterized by UV–vis spectroscopy (Shimadzu, Kyoto, Japan, UV-1800) and UV–vis DRS (Shimadzu, UV-2600). The Raman experiment was performed with confocal Raman microscopy (Dilor, France), and the laser excitation line was 632.8 nm with a power of 5 mW, equipped with semiconductor-cooled CCD detector. The Raman integral time for acquiring each spectrum was 8 s with three accumulations by using the laser confocal mode.

### 2.3. Fabrication of Poly(styrene-co-butadiene) Nanofiber

Poly(styrene-*co*-butadiene) was dissolved in a 1:4 (v/v) mixture of tetrahydrofuran and DMF to prepare an electrospinable solution (20 wt% SB). The polymer solution was stirred for 3 h until it was completely dissolved. The voltage difference between the needle (inner diameter: 0.5 mm) and the aluminum foil collector was set at 12 kV. The distance from tip to collector was about 15 cm in the experiment. The electrospinning process was carried out at a rate of 0.25 m/h.

### 2.4. Synthesis of Silver Nanoparticles

According to the conventional method [35], colloidal silver nanoparticles were prepared. Briefly, 0.0255 g of silver nitrate and 150 mL of ultrapure water were added into a flask, then boiled at about 90 °C for 10 min. Then, 3 mL of 1% (*w*/*w*) tri-sodium citrate solution was added. The solution was continuously boiled and stirred for 30 min until the color turned greyish-green.

### 2.5. Fabrication of Ag NPs on the SB Substrate

For fabrication of the electrospun, SB-based Ag NPs substrate, 10 μL of Ag NPs solution was dropped onto the electrospun SB substrate and dried at ambient temperature. After drying, 10 μL of the analyte was dropped on the flexible Ag/SB surface and then dried for the following SERS measurement.

### 2.6. SERS Analysis for Real Samples

Juice samples (10 μL) spiked with different concentrations of triazophos were dropped on the Ag/SB substrates and then dried at room temperature for SERS measurements.

To detect triazophos residue, 10 μL of triazophos solution was added on the peel of the apple and dried. Finally, the Ag/SB film was placed on the apple surface and pressed for 20 s and then taken to the Raman system to record SERS signals.

## 3. Results and Discussion

**Figure 1:**
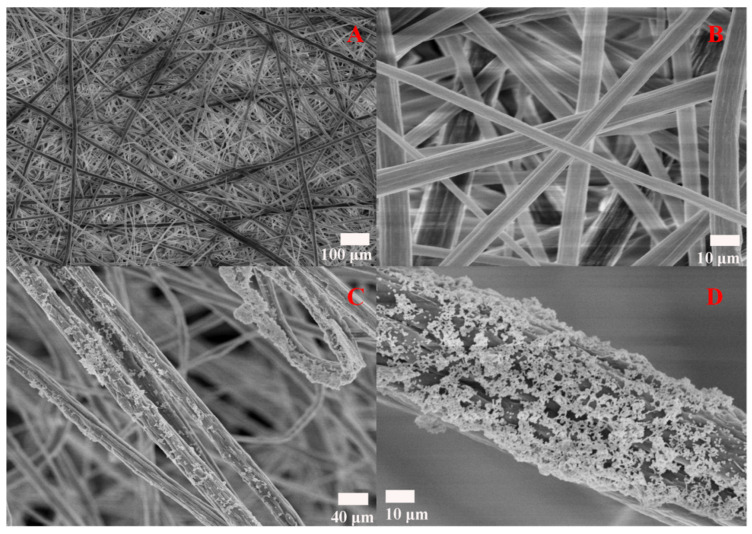
(**A**,**B**) SEM images of electrospun SB nanofibers, (**C**,**D**) SEM images of Ag NPs decorated SB nanofibers at different scales.

The preparation process of the Ag/SB film is illustrated in Supplementary Materials Figure S1. In brief, the SB nanofiber film was fabricated by electrospinning. Afterwards, silver colloids were deposited on the SB film surface. The full size of the Ag/SB membrane was around 1.5 cm × 1.5 cm with a thickness of about 2 mm. The morphologies of the electrospun SB and Ag/SB films were observed with FESEM. In Figure 1A,B, the SB film was composed of a large network framework. Figure 1C,D shows that colloidal Ag NPs were uniformly distributed on the surface of electrospun nanofibers. As shown in Figure S2, the contact angle of the Ag/SB film was about 130°, meaning the film was hydrophobic. In Figure S3, the morphology of the Ag NPs was spherical, the average size was about 60 nm, which exhibited certain aggregation of Ag NPs and generated many hot spots when dried.

**Figure 2:**
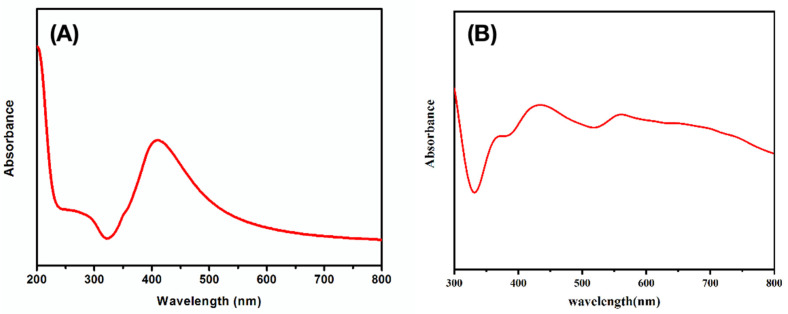
(**A**) UV–vis spectrum of Ag NPs colloidal solution. (**B**) UV–vis DRS of optimal Ag/SB film.

In Figure 2A, the UV–vis spectrum of the Ag colloid presents a strong surface plasmon resonance band around 410 nm. By contrast, in Figure 2B, the UV–vis diffuse-reflectance spectrum (DRS) of Ag/SB film has a broad surface plasmon resonance band due to the suitable aggregation of Ag NPs, which agrees with the SEM observations. As a result, the hydrophobic Ag/SB film with nonplanar Ag NPs loading in homogeneity provided more “hot-spot” to benefit SERS detection.

For comparison, SEM images of Ag NPs on filter paper are also displayed in Figure S4. Any inhomogeneity of Ag NPs distribution on filter paper is not conducive to obtaining good signals in the following Raman experiments.

### 3.1. Optimization of Ag NPs Usage

**Figure 3:**
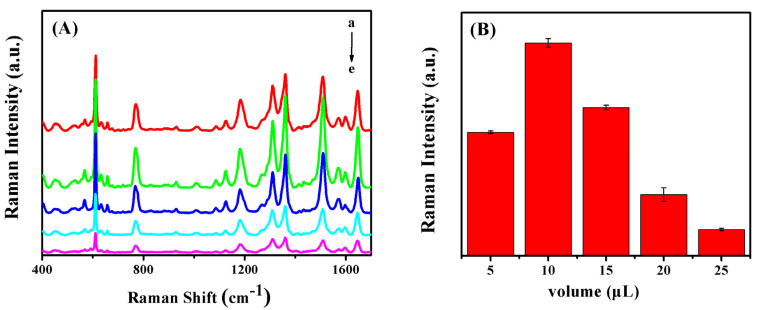
(**A**) SERS spectra of R6G (10 μL, 1 μM) acquired on Ag/SB nanofibers, prepared by dropping different amounts of silver colloidal, (a) 5 μL, (b) 10 μL, (c) 15 μL, (d) 20 μL, (e) 25 μL. (**B**) The corresponding bars of SERS peak intensities at 1510 cm^−1^.

**Figure 4:**
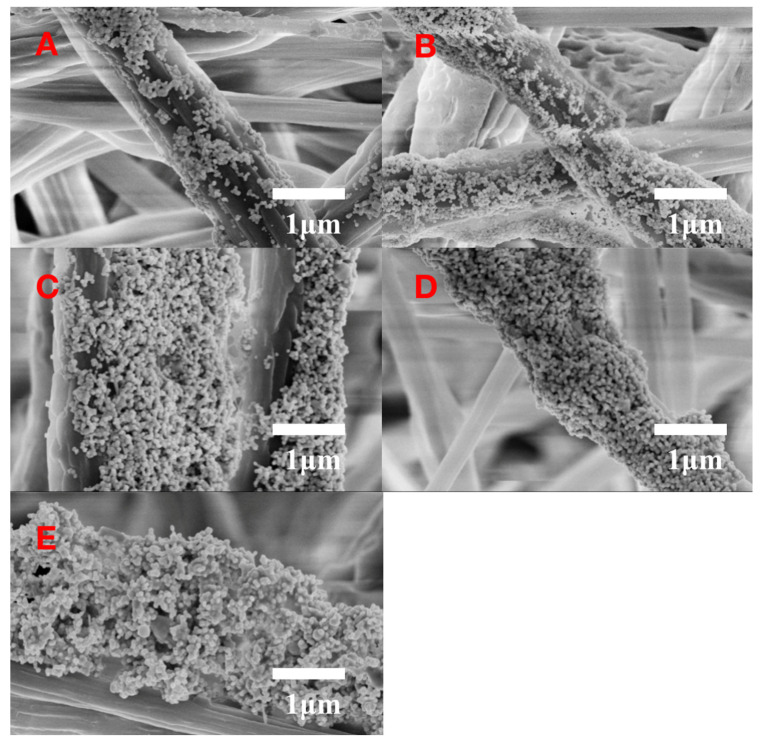
SEM image of Ag/SB nanofibers, prepared by dropping different volumes of silver colloid, (**A**) 5 μL, (**B**) 10 μL, (**C**) 15 μL, (**D**) 20 μL, (**E**) 25 μL.

The usage of colloidal Ag NPs was optimized to acquire the highest SERS signals by using 1 μM R6G solution as the Raman probe. Figure 3A shows the SERS spectra of R6G recorded with Ag/SB films prepared by adding 5, 10, 15, 20, and 25 μL of Ag NPs colloid solutions. According to the histogram in Figure 3B, it could be confirmed that the Ag/SB film made with 10 μL of Ag colloid solution had the optimal SERS performance. By deposition of different volumes of Ag colloid on the SB film, FESEM images were taken. As shown in Figure 4, depositing 10 μL of Ag colloid led to a relatively uniform distribution of Ag NPs on the SB fibers. By contrast, the usage of Ag colloid more than 10 μL resulted in notable aggregation, while Ag colloid less than 10 μL resulted in less Ag NPs on the surface of the SB film.

### 3.2. SERS Performance of Ag/SB Film

**Figure 5:**
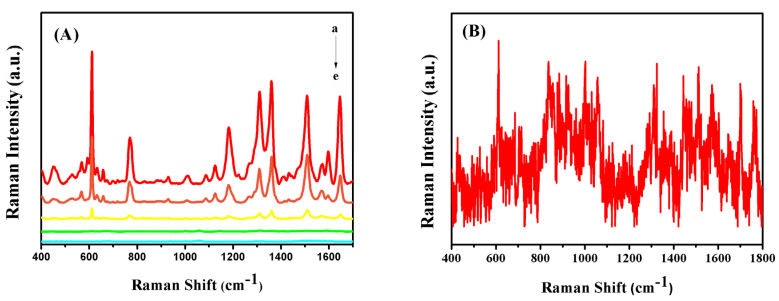
(**A**) SERS spectra of R6G with different concentrations on Ag/SB substrate, (a) 1 μM, (b) 100 nM, (c) 10 nM, (d) 1 nM, (e) blank. (**B**) The enlarged SERS spectrum recorded on Ag/SB substrate with 1 nM R6G.

R6G was employed to quantitatively evaluate the Raman scattering enhancement effect of the optimal Ag/SB film. The concentration-dependent SERS spectra of R6G are given in Figure 5A and clearly seen in Figure 5B. When the concentration of R6G was down to 10^−9^ M, a Raman peak at 611 cm^−1^ could be identified. This proved the Ag/SB film was greatly enhanced and could be applied to analyze trace samples.

For checking the stability of the Ag/SB film, UV–vis DRS was carried out on the substrate before and after laser detection. In Figure S5, a similar surface plasmon resonance band of Ag NPs can be observed in comparison with DRS in Figure 2B, indicating that laser exposure did not influence the Ag/SB film. Additionally, in this work, Ag/SB films were easily and reproducibly prepared on a large scale and could be used as disposable SERS substrates. In Figure S6, the small Raman signal from the SB film means that the background signals would not affect the detection of target sample.

For comparison, as mentioned above, a filter paper Ag NPs substrate was also prepared. In SEM images, the hydrophilic filter paper hardly concentrated Ag NPs on the small region, resulting in fewer hot spots. As depicted in Figure S7, by using a filter paper Ag NPs substrate, Raman signals of 10^−6^ M R6G were hardly visible. Consequently, the Raman detection sensitivity by using the Ag/SB film was 10-fold higher than the hydrophilic one.

For estimating the flexibility of Ag/SB, after folding it 100 times, 1 μM R6G was added onto the Ag/SB substrate to record the Raman spectrum. The photograph is given in Figure S8, and no obvious change in shape was found. In Figure S9, SERS signals of R6G obtained on the Ag/SB film, folded 100 times, showed a slight change as compared with Raman signals recorded for the freshly prepared Ag/SB film. This demonstrated the flexible Ag/SB film is capable of detecting analytes on-site or in the field after sampling in a wiping way.

**Figure 6:**
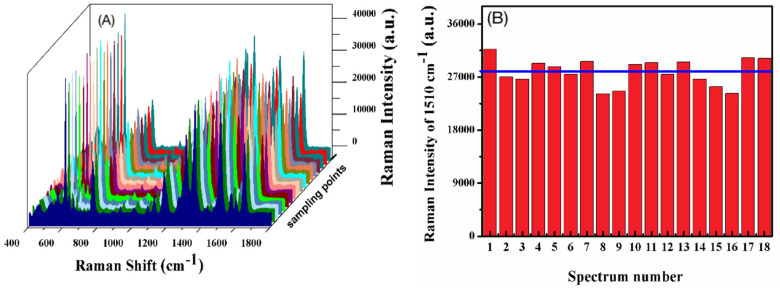
(**A**) Reproducibility of SERS signals of 1-μM R6G on Ag/SB films, recorded from 18 randomly selected spots. (**B**) SERS intensity distribution estimated on the SERS peak at 1510 cm^−1^.

**Figure 7:**
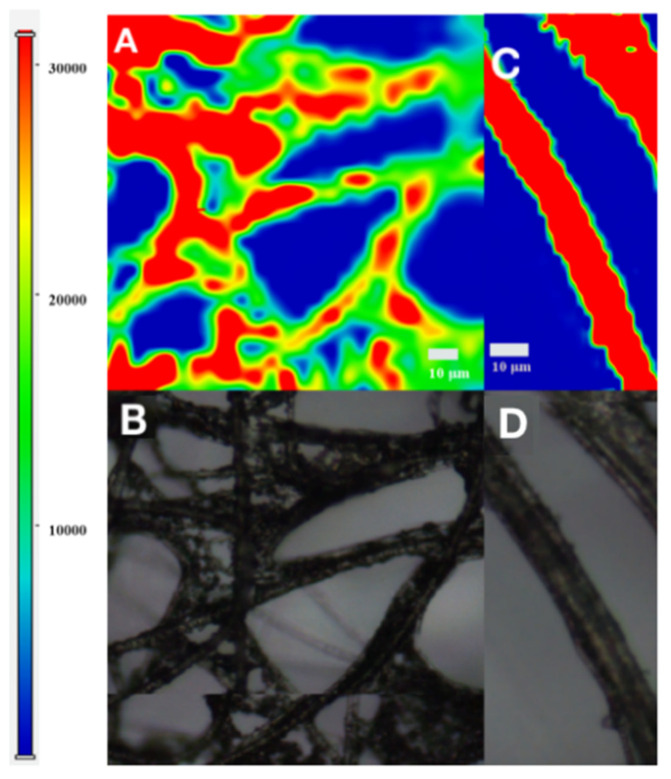
(**A,C**) SERS surface mapping image of 1 μM R6G on Ag/SB film of the selected region, (**B**,**D**) the bright light picture corresponding to (**A**,**C**) respectively, using the intensity at 611 cm^−1^.

For investigating the uniformity of the Ag/SB film, we chose 18 random points on the surface to detect a SERS signal of 1 μM R6G (Figure 6A). The statistic bars in Figure 6B are based on the Raman intensity at 1511 cm^−1^ with a calculated relative standard deviation (RSD) of about 8.12%, which remarks a reasonable reproducibility, attributed to the hydrophobic SB film preventing Ag NPs from randomly diffusing and presenting the well-dispersed state. Similarly, SERS mapping was also utilized to observe the distribution of SERS signals on the Ag/SB film. As shown in Figure 7, good uniformity of SERS on the Ag/SB film could be visualized.

**Figure 8:**
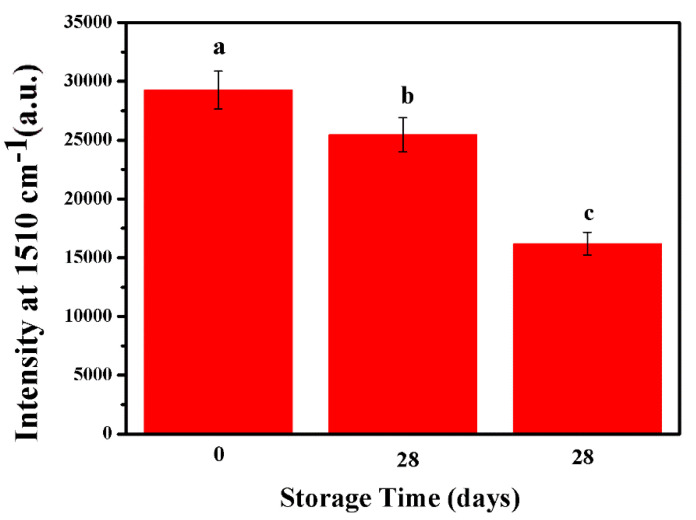
SERS spectra of R6G (10^−6^ M) recorded on different Ag/SB films, stored within (a) 0 day, (b) 28 days in N_2_ atmosphere, and (c) 28 days in air atmosphere.

The stability of the optimal Ag/SB film had been monitored for 28 days by storage in ambient and N_2_/4 °C conditions. In Figure 8, Ag/SB film stored in N_2_ at 4 °C showed 87.21% of the original Raman intensity after 28 days, which had better stability than that kept in an ambient atmosphere (55.3% recovery of signal after 28 days). Consequently, the long shelf-time of Ag/SB film in N_2_/4 °C could meet the requirement of real sample detection.

### 3.3. SERS Measurement of Triazophos

Triazophos is an organophosphate pesticide (Ops), and its residue in food has been a concern in European food surveillance programs [36]. The passivated acetylcholinesterase (AChE) activity in the nervous system could be responsible for the highly toxic levels of Ops [37]. Therefore, triazophos residue on fruits, water, and crops will bring immeasurable damage to human health via the food chain [38], particularly for children. Various methods for detection of triazophos have been explored, including HPLC [39], enzyme-based electrochemistry [40], and GC [41], but such methods suffer from tedious sample pretreatment and require well-experienced technicians. Yan et al. set up a triazophos colorimetric and SERS method via proposing a biomimetic nanozyme-linked immunosorbent assay together with a molecular imprints technique [42], but the synthesis method of such substrate was complicated and time-consuming.

**Figure 9:**
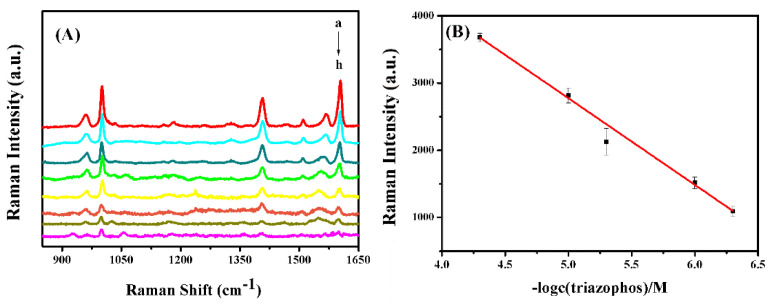
(**A**) Concentration-dependent SERS spectra of triazophos on the Ag/SB film, from a to h: 50 μM, 10 μM, 5 μM, 1 μM, 500 nM, 100 nM, 50 nM, and 25 nM. (**B**) Calibration linear plot from 5 × 10^−5^ to 5 × 10^−7^ M based on Raman intensity at 1406 cm^−1^ (R^2^ = 0.997).

Herein, an optimal Ag/SB film was used to detect the triazophos. As displayed in Figure 9A, the main Raman bands for triazophos at 1001, 1406, and 1604 cm^−1^ can be identified, which are roughly the same as the Raman spectra reported [43,44,45] previously, and the intensity increased with elevating concentration of triazophos. In Figure 9B, a linear dynamic concentration relationship is plotted between 5 × 10^−5^ to 5 × 10^−7^ M with R^2^ = 0.997 based on the intensities at 1406 cm^−1^. As mentioned above, the hydrophobic Ag/SB film could drag triazophos molecules approaching the hot spot region during a slow drying. Through this, the signal-to-noise (S/N) ratio was equal to 3, as seen in Figure 9A, and a LOD of about 2.5 × 10^−8^ m could be reached.

It should be stated that, by using an SERS substrate based on filter paper Ag NPs, the triazophos could be detectable when the concentration was at 10^−4^ M (Figure S10).

**Figure 10:**
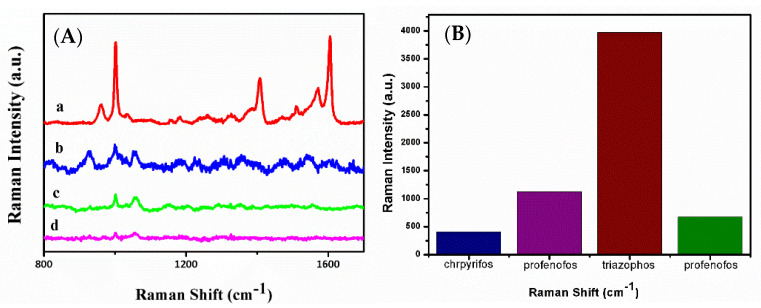
(**A**) SERS spectra of some pesticide interferences (5 × 10^−6^ M) on Ag/SB film: (a) triazophos, (b) profenofos, (c) parathion, (d) chrpyrifos. (**B**) The visualized bar graph of SERS detection selectivity of triazophos on Ag/SB film, and the concentrations for other pesticides are 5 × 10^−6^ M.

As shown in Figure 10, the effect of the above pesticides, including profenofos, parathion, and chrpyrifos, on Raman detection of triazophos could be ignorable. Thus, the detection selectivity for triazophos by using an Ag/SB-based SERS assay is validated.

### 3.4. SERS Detection of Triazophos in/on Real Samples

Without any sample preparations, triazophos residue in apple juice and on apple peels were detected by a Ag/SB-based Raman method, and the results are tabulated in Table 1 and Table 2. In the cases of apple juice spiked with 50, 10, and 5 μM of triazophos, the recoveries ranged from 85% to 106%, while for 50, 10, and 5 μM triazophos residues on apple peels, the recoveries were located in the range from 87% to 102%.

HPLC measurements of triazophos in juice were also performed for investigating the reliability of the Ag/SB-based SERS method. HPLC results (Table 2) further confirmed the robustness of this proposed SERS method as well as the sensitivity and rapidness for SERS inspection of triazophos in food.

As listed in Table 3, the Ag/SB-based SERS method had a notably better result regarding the detection limit and recovery compared to other methods in the literature. It should be emphasized that, in this work, the electrospun SB film could be obtained in a scalable way.

Furthermore, the resistance of Ag/SB-film to heat, due to both its plastic-like rigidity and rubber-like elasticity, could also be explored to meet the needs of specific detection aims. As an example, the anticancer drug 6-mercaptopurine (6MP), which dissolves in hot water, precipitates at ambient temperatures, which would negatively affect the accuracy of SERS determination. As clearly seen in Figure S11, the SERS signal intensity recorded from the 6MP solution around 90 °C was greater than that at room temperature (25 °C), simultaneously remarking that the Ag/SB film has excellent heating resistance.

## 4. Conclusions

The flexible and hydrophobic Ag/SB nanofiber films as SERS substrates were fabricated through electrospinning technology in a large-scale and reproducible way. By using R6G as probe molecule, a high sensitivity and signal reproducibility of the Ag/SB-SERS method, as well as a high stability of the substrate, were observed. Without complicated sample preparation, by using an Ag/SB-Raman approach, LOD of Raman analysis of triazophos could be reached at 2.5 × 10^−8^ M. Since the Ag/SB film showed good heat durability, it could be used to conduct SERS detection of specific samples in hot water. Due to the promising merits of electrospun Ag/SB film such as its good synthesis reproducibility, low cost, and large-scale production, Ag/SB-based SERS protocol is a desired platform to analyze hydrophobic pesticide residue or organic contaminants.

## Figures and Tables

**Table 1:** Determination of triazophos on apple peel by the proposed Ag/SB-based SERS method.

Samples	Added (μM)	Detected (μM)	Recovery Rate (%)	RSD (%)
Apple peel	50	51	102	7.6
	10	8.7	87	7.7
	5	4.8	96	7.0

**Table 2:** Determination of triazophos in apple juice by Ag/SB-based SERS and HPLC methods.

Samples	Added (μM)	SERS (μM)	SERS Recovery (%)	HPLC (μM)	HPLC Recovery (%)
Apple juice	50	49	98	49	98
	10	8.5	85	9.1	91
	5	5.3	106	4	80

**Table 3:** Comparison of detection performance of triazophos by Ag/SB-film-based SERS with other methods.

Method	Nanomaterial	Detection Limit (M)	Recovery (%)	References
Colorimetry	MPA-GAA-Ag NPs	5 × 10^−7^	92.18–99.64	[46]
FPIA	-	10^−7^	76–110	[47]
SERS	PDMS-Ag NPs	10^−7^	-	[48]
HPLC-FLD	-	6 × 10^−8^	86.3–104.2	[49]
SERS	Ag/SB-film	2.5 × 10^−8^	85–106	This work

## Supplementary Materials

The following are available online at https://www.mdpi.com/1424-8220/20/15/4120/s1, Figure S1: The fabrication of the SERS substrate and its application for extracting of target sample from fruit peel surface (apple). Figure S2: The water contact angle of the electrospun SB film. Figure S3: SEM image of Ag/SB film. Figure S4: SEM images of Ag NPs decorated filter paper with different scales. Figure S5: UV–vis DRS of R6G (1 μM) recorded on Ag/SB film. Figure S6: Raman spectrum of electrospun SB film. Figure S7: SERS spectra of R6G with different concentrations on filter-paper-based Ag NPs substrate: (a) 10 μΜ, (b) 1 μΜ. Figure S8: Photograph of Ag/SB film (a) before (b) after folding for 100 times. Figure S9: SERS spectra of 10 μL, 1 μΜ of R6G on Ag/SB film. (a) Unfolding (b) After folding times for 100. Figure S10: SERS spectra of triazophos with different concentrations on filter-paper-based Ag NPs substrate: (a) 10^−3^ M, (b) 10^−4^ M. Figure S11: SERS spectra of 10^−2^ M 6-MP on Ag/SB film at (a) temperature at 90 °C (b) room temperature.
