# Supplementary material for: Preparation of Hydrophobic Film by Electrospinning for Rapid SERS Detection of Trace Triazophos
Source: Sensors (Basel). 2020 Jul 24;20(15):4120. doi: 10.3390/s20154120 (PMC7436116; doi:10.3390/s20154120)
Supplement: Supplementary file 1 [file sensors-20-04120-s001.pdf]

Supplementary Material

Preparation of Hydrophobic Film by Electrospinning for Rapid SERS Detection of Trace Triazophos

Fei Shao[#], Jiaying, Cao[#], Ye Ying, Ying Liu, Dan Wang, Xiaoyu Guo, Yiping Wu, Ying Wen, Haifeng Yang*

The Education Ministry Key Lab of Resource Chemistry, Shanghai Key Laboratory of Rare Earth Functional Materials, Shanghai Municipal Education Committee Key Laboratory of Molecular Imaging Probes and Sensors and Department of Chemistry, Shanghai Normal University, Shanghai 200234, China

# These authors contributed equally to this work.

**Corresponding Authors*

Telephone: +86-21-64321701. E-mail: hfyang@shnu.edu.cn (Haifeng Yang), yingye@shnu.edu,cn (Ying Ye)

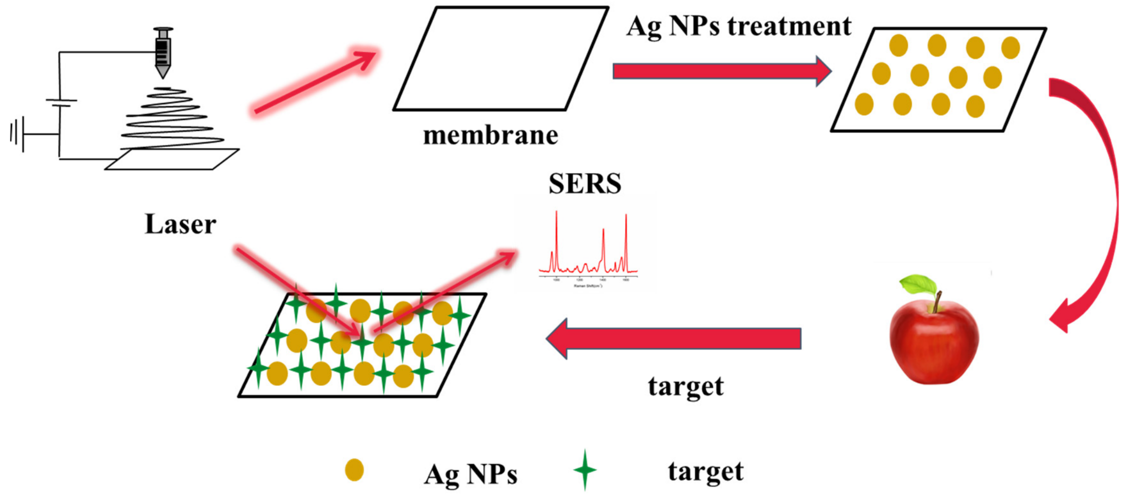

Figure S1. The fabrication of the SERS substrate and its application for extracting of target sample from fruit peel surface (apple).

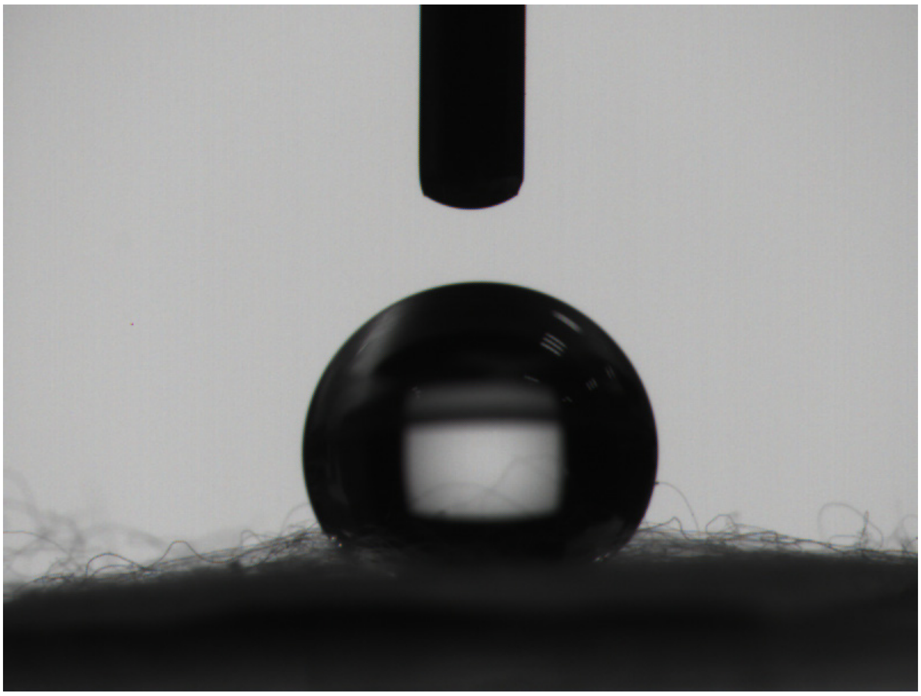

Figure S2. The water contact angle of the electrospun SB film.

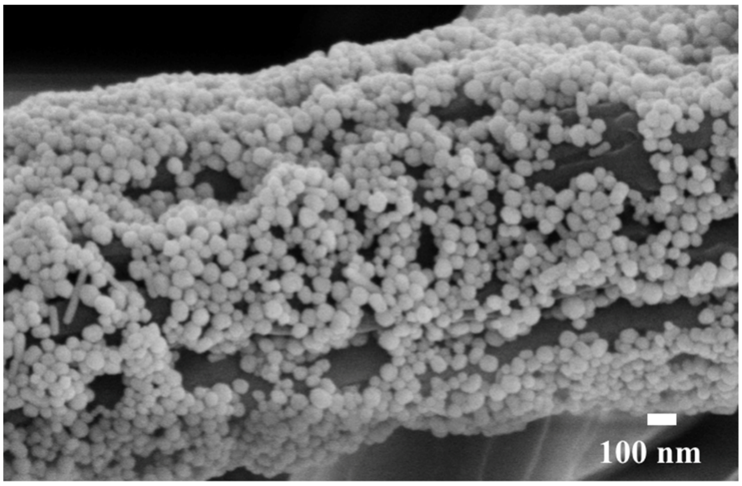

Figure S3. SEM image of Ag/SB film

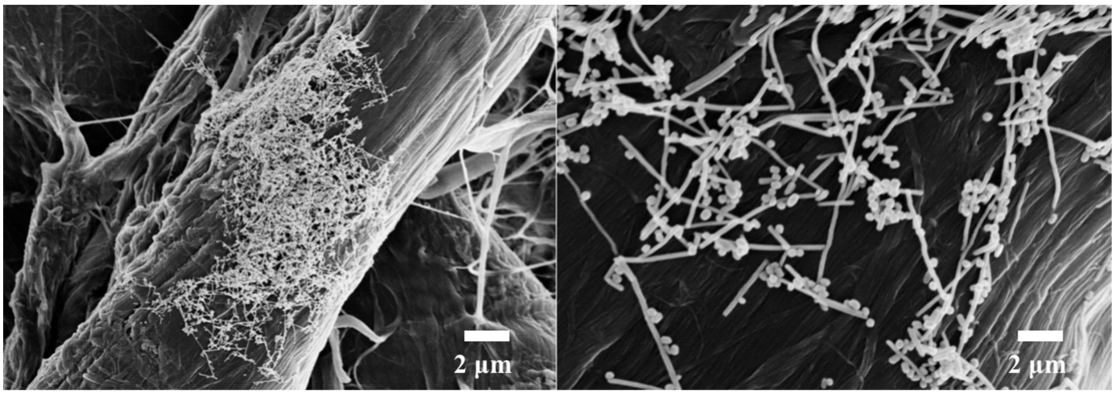

Figure S4. SEM images of Ag NPs decorated filter paper with different scales.

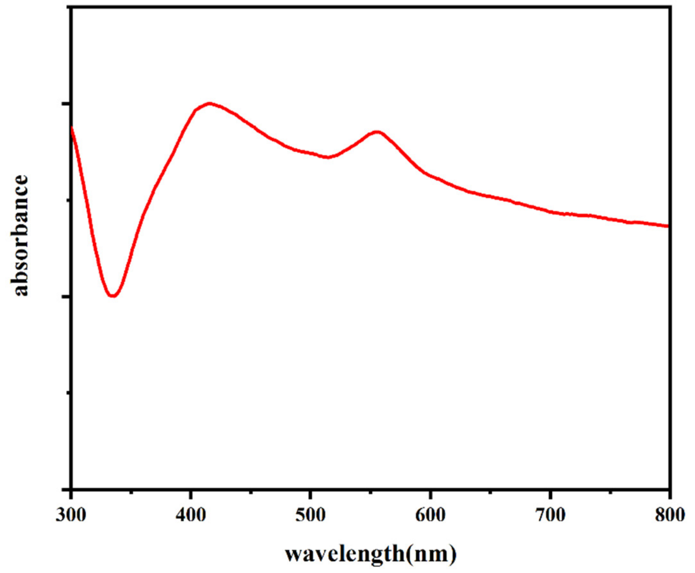

Figure S5. UV–vis DRS of R6G (1 μM) recorded on Ag/SB film.

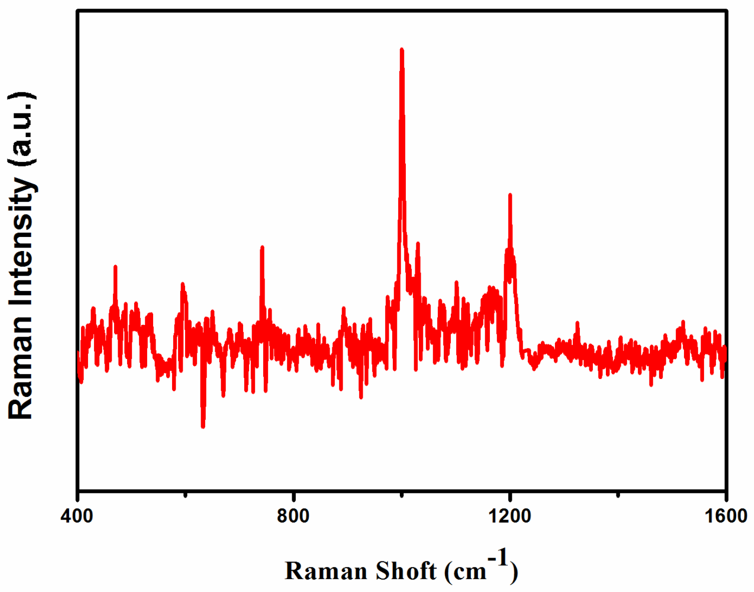

Figure S6. Raman spectrum of electrospun SB film.

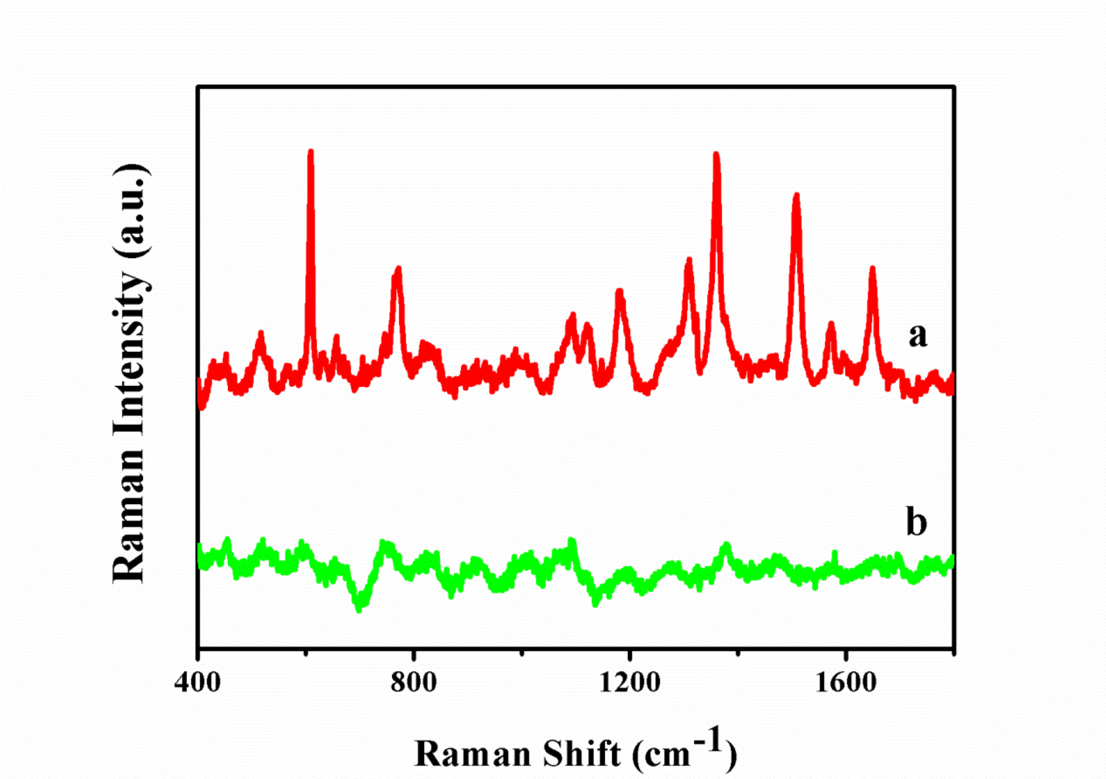

Figure S7. SERS spectra of R6G with different concentrations on filter-paper-based Ag NPs substrate: (a) 10 μM, (b) 1 μM.

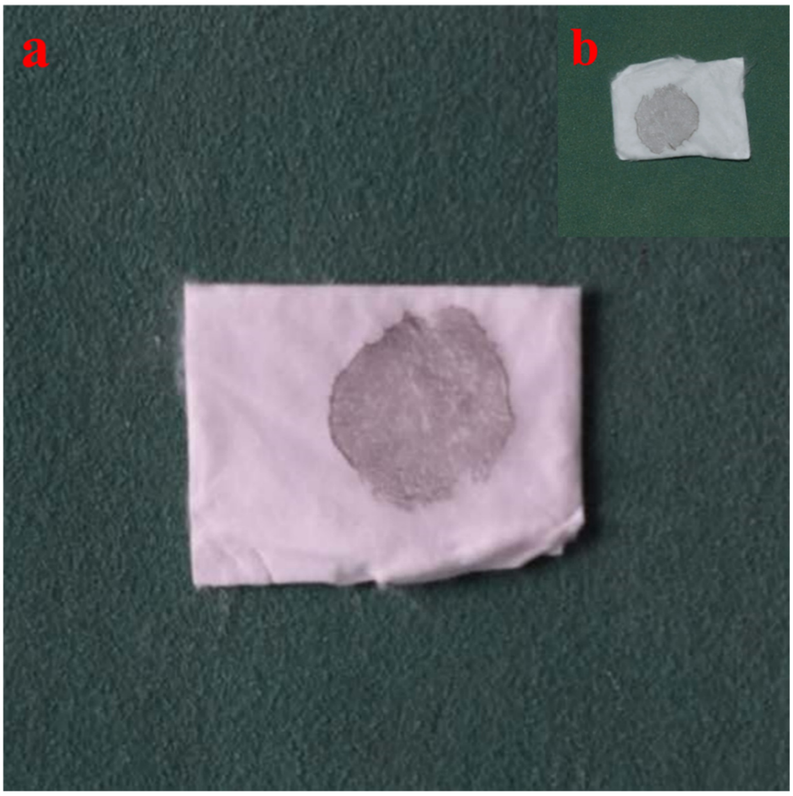

Figure S8. Photograph of Ag/SB film (a) before (b) after folding for 100 times.

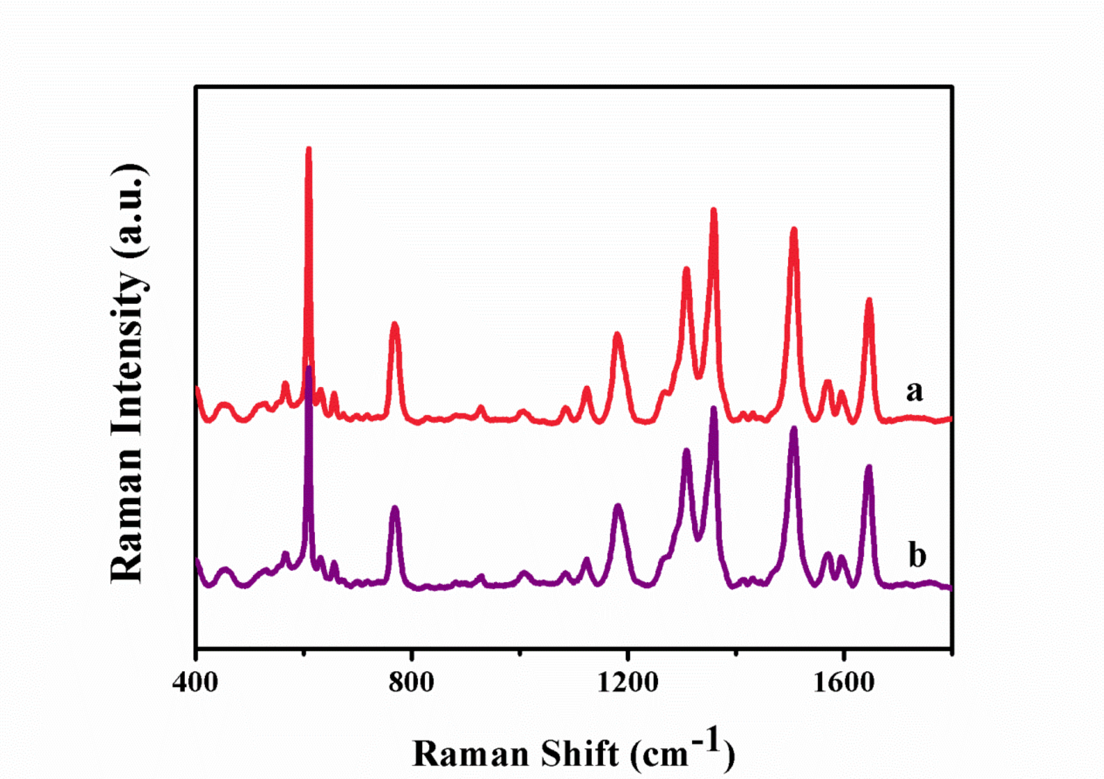

Figure S9. SERS spectra of 10 μL, 1 μM of R6G on Ag/SB film. (a) Unfolding (b) After folding times for 100.

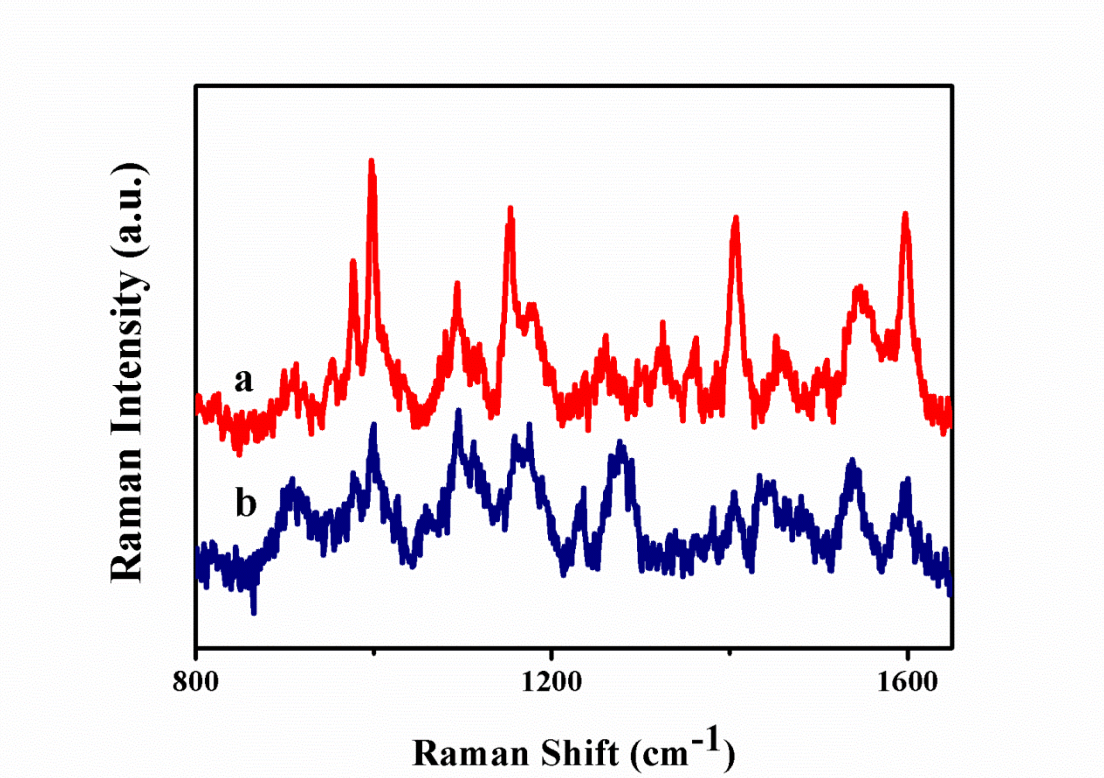

Figure S10. SERS spectra of triazophos with different concentrations on filter-paper-based Ag NPs substrate: (a) 10^{-3} M, (b) 10^{-4} M.

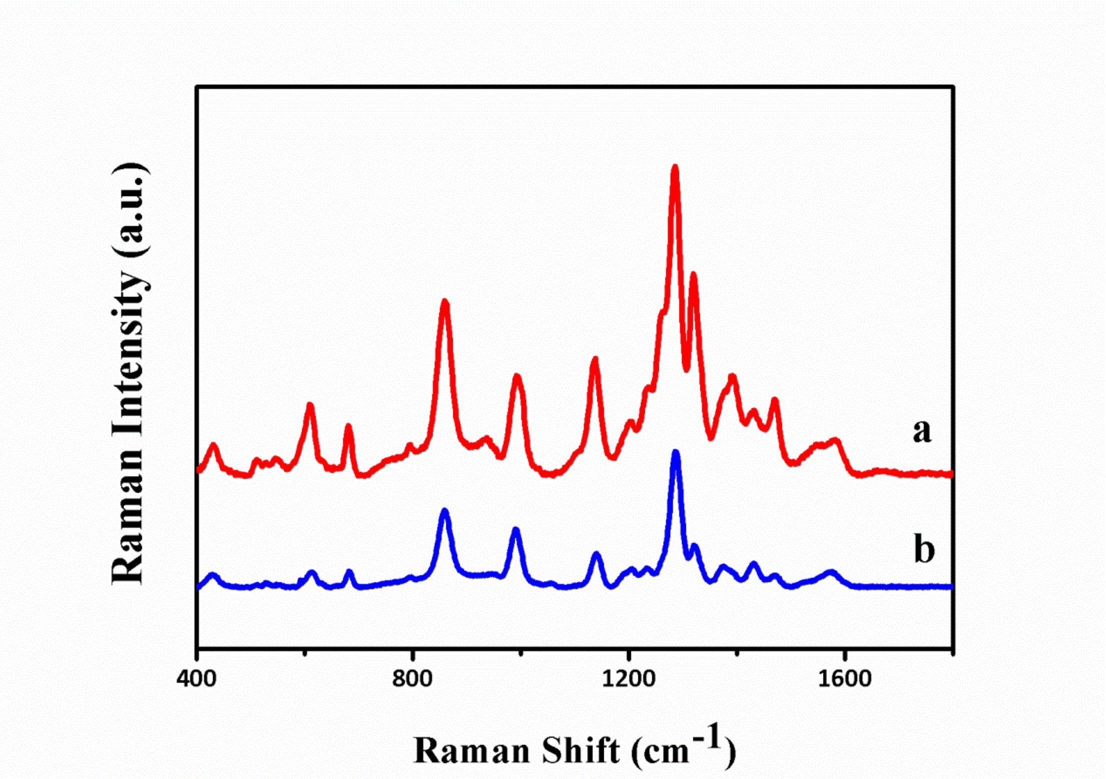

Figure S11. SERS spectra of 10^{-2} M 6-MP on Ag/SB film at (a) temperature at 90 °C (b) room temperature
